# Updating the Role of Carboplatin Added to Neoadjuvant Chemotherapy in Early Triple-Negative Breast Cancer: A Meta-Analysis

**DOI:** 10.3390/cancers17243961

**Published:** 2025-12-12

**Authors:** Ida Taglialatela, Beatrice Ruffilli, Benedetta Conte, Francesca D’Avanzo, Valentina Rossi, Simone Nardin, Alessandra Gennari

**Affiliations:** 1 AOU Maggiore della Carità, 28100 Novara, Italy; 2 Department of Translational Medicine, University of Eastern Piedmont, 28100 Novara, Italy

**Keywords:** triple-negative breast cancer, carboplatin, neoadjuvant therapy

## Abstract

Background: Triple-negative breast cancer (TNBC) is associated with poor prognosis and a high risk of early relapse. The incorporation of platinum-based agents into neoadjuvant chemotherapy (NACT) regimens has been linked to improved pathological complete response (pCR) rates. However, the clinical benefit of carboplatin (CBDCA) remains debated due to variable long-term survival outcomes and concerns over cumulative toxicity. This meta-analysis evaluates the efficacy of adding CBDCA to NACT in early-stage TNBC (eTNBC). Methods: A systematic review and meta-analysis were conducted by searching MEDLINE, PubMed, and major oncology conference proceedings (2014–2024), with no language restrictions. Randomized phase II–III trials assessing the addition of CBDCA to standard NACT in eTNBC and reporting pCR and survival outcomes were included. The systematic review followed the recommendations of the Preferred Reporting Items for Systematic Reviews and Meta-Analyses (PRISMA). The protocol has not been registered. The primary endpoint was pCR; the secondary endpoint was disease-free survival (DFS). For pCR, a random-effects model was used, and odds ratios (OR) were log-transformed. For DFS, a mixed-effects model was applied, extracting hazard ratios (HR) and converting them into logHR values. Heterogeneity was assessed using I^2^ statistics, and publication bias was evaluated through the Fail-Safe N method and Egger’s regression test. Statistical analyses were performed using Jamovi v2.4.11. Results: Of 30 studies identified, 9 randomized clinical trials were eligible; 6 (BrighTNess, GeparSixto, GS5-01, BR-15-1 PEARLY, NACATRINE, CALGB 40603) met all inclusion criteria, totaling 3402 patients. The addition of CBDCA to NACT significantly improved pCR (OR 1.63; 95% CI: 1.38–1.92; *p* < 0.001), with low heterogeneity (I^2^ = 0.81%) and no publication bias. DFS was also significantly improved (SHR 0.81; 95% CI: 0.63–0.91; *p* = 0.003), with moderate heterogeneity (I^2^ = 27.95%) and no bias detected. Conclusions: Adding carboplatin to NACT significantly improves pCR and DFS in patients with early-stage TNBC.

## Simple Summary

Triple-negative breast cancer is an aggressive subtype associated with poor prognosis and limited targeted treatment options. The addition of carboplatin to standard neoadjuvant chemotherapy is proposed to enhance treatment efficacy; however, its overall clinical benefit and potential toxicity remain subjects of debate. This meta-analysis aimed to clarify the role of carboplatin in the management of early-stage triple-negative breast cancer by integrating evidence from randomized clinical trials. The pooled results demonstrate that incorporating carboplatin significantly increases the rate of pathologic complete response and improves disease-free survival. These findings provide strong support for the inclusion of carboplatin in neoadjuvant treatment regimens and contribute valuable evidence to guide clinical decision-making and future research in this challenging disease setting.

## 1. Introduction

Triple-negative breast cancer (TNBC), accounting for 10–20% of breast cancer cases, is typically diagnosed at a younger age and is more often associated with *BRCA 1* and *BRCA* 2 germline pathogenic variants [1]. Because it is considered a heterogeneous subtype with a poor prognosis due to a higher risk of relapse, treatment standards have evolved significantly over the years.

The rationale of using platinum salts in triple-negative breast cancer is sustained by biological reasons. Specifically, a great number of *BRCA* mutant breast tumors are triple-negative and could benefit from the synthetic lethality offered by carboplatin due to their defective homologous recombination repair (HRR), conferring them a particular susceptibility to the carboplatin mechanism of action. Platinum salts work by inducing DNA crosslinks and double-strand breaks, and cells with defective homologous recombination repair are killed more easily than proficient HRR cells. This aligns Carboplatin’s mechanism with TNBC biology [2].

Beyond the biological aspects, a real confirmation comes from the clinical evidence in the early stage, both in the neoadjuvant and the adjuvant setting. Despite the standard use of anthracycline and taxane as neoadjuvant chemotherapy, the substitution of anthracycline with carboplatin implied a significant increase in pCR cases in the NeoCART trial, with 61.4% of pCR in the experimental arm versus 38.6% in the standard arm [3]. The benefit of using carboplatin also regards the event-free survival, as shown in the PEARLY trial with a benefit both in the pCR rate (45.6% vs. 39.3%) and in EFS (HR 0.68, 95% CI 0.5–0.9) [4]. These effective data are confirmed by the meta-analysis of Poggio and colleagues, in which the pooled analysis of randomized trials confirmed this finding (RR 1.44, 95% CI 1.27–1.63; *p* < 0.001) [5].

Platinum salts also have a role in the adjuvant setting. As shown in the PATTERN phase III trial, a multicentric randomized trial comparing adjuvant therapy with paclitaxel and carboplatin versus cyclophosphamide/Epirubicin/5-FU/Docetaxel in 647 triple-negative breast cancer patients after surgery, a benefit in DFS was observed for the experimental arm with carboplatin (HR 0.65, 95% CI 0.44–0.96) [6].

Pathological complete response (pCR) after neoadjuvant chemotherapy is a strong prognostic marker at the patient level, being consistently associated with improved event-free and overall survival, particularly in biologically aggressive subtypes such as triple-negative and HER2-positive breast cancers [7,8]. However, at the trial level, the largest pooled analysis to date (CTNeoBC) failed to validate pCR as a surrogate endpoint for long-term outcomes across all breast cancer subtypes, indicating that increases in pCR rates do not necessarily translate into proportional improvements in survival between treatment arms [7]. Consequently, pCR cannot be considered a universal surrogate endpoint for regulatory purposes.

The U.S. Food and Drug Administration has nonetheless recognized that an improvement in pCR “could be reasonably likely to predict long-term benefit” in high-risk early breast cancer, allowing accelerated approval in specific contexts provided that confirmatory data on event-free or overall survival are subsequently obtained [9]. More recent systematic reviews and meta-analyses have reaffirmed the strong patient-level prognostic value of pCR, especially in triple-negative breast cancer, while highlighting ongoing debate regarding its validity as a trial-level surrogate, as results vary depending on study design, patient population, and pCR definition [10].

The incorporation of carboplatin into neoadjuvant or adjuvant chemotherapy regimens for triple-negative breast cancer (TNBC) is consistently associated with increased hematologic toxicity compared with standard anthracycline–taxane combinations. Meta-analyses and randomized trials have reported higher incidences of grade 3–4 neutropenia and thrombocytopenia, often resulting in increased rates of dose modifications and treatment delays [5,6,7,8,9,10,11]. Despite these effects, toxicity is generally manageable with appropriate supportive measures, including growth factor support and careful monitoring, and rarely leads to permanent treatment discontinuation [12]. From a broader tolerability perspective, anthracycline-sparing regimens such as paclitaxel–carboplatin may offer advantages in long-term cardiac safety and cumulative toxicity, particularly for younger patients or those with pre-existing cardiovascular risk factors [6]. Ongoing studies are also evaluating alternative dosing strategies, including weekly versus every-three-week carboplatin schedules, to optimize the balance between efficacy and tolerability [13,14,15]. Overall, while hematologic adverse events are more frequent with carboplatin-containing regimens, these are counterbalanced by potential reductions in non-hematologic toxicities and favorable long-term safety profiles when anthracyclines are omitted.

Current guidelines recommend to use of neoadjuvant chemotherapy (NACT) for eTNBC with a primary tumor with a diameter > 1 cm and/or presence of metastatic loco-regional lymph nodes, in order to minimize the probability of developing distant metastases and to allow conservative surgery [16,17].

Specifically, the standard regimen includes platinum-based chemotherapy with taxanes and anthracyclines, and for high-risk eTNBC, the addition of immune checkpoint inhibitors before and after surgery, based on the positive results of the KEYNOTE-522 trial in both RFS and OS for high-risk eTNBC [18].

Numerous studies and meta-analyses also demonstrate the benefits of adding carboplatin to taxane and anthracyclin-based chemotherapy in the neoadjuvant setting for these patients, primarily in terms of pCR rate (an increase of up to 20%) [19,20,21]. Despite including carboplatin in the standard of care, the true impact of this modification on long-term outcomes and patients’ quality of life remains controversial. In fact, severe and life-threatening hematological adverse events were significantly more common in patients receiving platinum-based neoadjuvant chemotherapy, sometimes leading to early discontinuation, dose modifications, or skipped doses, given the uncertain benefit in overall survival and relapse-free survival [5].

The pathological response acquired an impactful role as prognostic biomarker due to the evidence offered by the CTNeoBC pooled analysis of neoadjuvant breast cancer clinical trials, suggesting that patients achieving a pathologic complete response (pCR) had a significantly better prognosis than those who had residual disease, and this association was strongest in patients with triple-negative and HER2–positive disease [7,8].

Consequently, the achievement of pathological complete response (pCR), defined as the absence of invasive residual disease in the breast of lymph nodes, acquired a special prognostic value and new more refined scores seem to be useful in the cases who received chemo-immunotherapy, such as RCB index (Residual Cancer Burden index), calculated by considering dimensions of the primary tumour, tumour bed cellularity and axillary nodal burden [22,23].

Despite the chemosensitivity of TNBC, only 30–40% of patients achieve a pCR with anthracycline–taxane-based chemotherapy, and recent evidence shows the benefit of adding carboplatin to NACT in eTNBC, leading to improved pCR rates [23]. This could increase the chances of achieving pCR compared to NACT schedules without platinum salts for g*BRCA*-PV eTNBC patients, due to their higher sensitivity to platinum salts, as suggested by considerable evidence [24]. In fact, the impact of immunotherapy in this group of patients remains unknown because the KEYNOTE-522 trial did not include stratification or subgroup analysis.

Building on previous evidence and aiming to update this topic, we conducted a systematic review and meta-analysis of the literature on the role of adding carboplatin to neoadjuvant chemotherapy in early TNBC, seeking to update current data on platinum effects in the neoadjuvant setting.

## 2. Materials and Methods

A systematic review and meta-analysis were conducted by searching MEDLINE, PubMed and the principal oncology meetings articles and abstracts from 2014 to 2024 with no restriction of language.

The research was performed through MeSH terms using the following search string: ((“breast cancer” OR “breast carcinoma” OR “mammary carcinoma”) AND (“neoadjuvant therapy” OR “neoadjuvant treatment” OR “preoperative therapy”) AND (“carboplatin”) AND (“randomized controlled trial” OR “RCT” OR “randomised controlled trial”) AND (“phase II” OR “phase III”) AND (“2014/01/01”[PDAT]: “2024/12/31”[PDAT]) AND (clinical trial[pt] OR randomized controlled trial[pt]) AND (adult[MeSH Terms])).

Phase II-III randomized studies evaluating the addition of carboplatin to neoadjuvant chemotherapy (NACT) in early Triple-Negative Breast Cancer (eTNBC) reporting response rate and survival outcomes were included. Case reports, reviews and observational studies were excluded. In detail, starting with 30 studies screened, we excluded studies with a follow-up shorter than 3 years, lacking data on pCR rate and EFS or DFS rate, without a clear methodology, or with a control arm using a treatment regimen different from the standard, which includes anthracycline, cyclophosphamide, and taxane (i.e., FEC or ET regimen).

**Figure 1:**
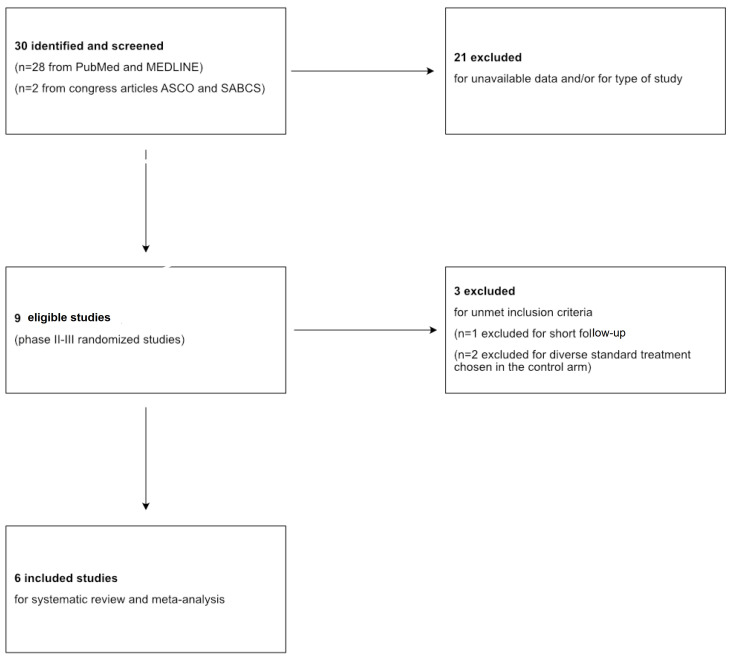
PRISMA flowchart of search results and study selection.

The systematic review followed the recommendations of the Preferred Reporting Items for Systematic Reviews and Meta-Analyses (PRISMA). The protocol has not been registered. Figure 1 represents a flow diagram of the study selection.

Pathological complete response (pCR) was the primary endpoint; disease-free survival (DFS) was the secondary endpoint of our investigation.

For the primary endpoint (pCR), a random-effects model was used for data analysis, whereas the Odds Ratio (OR) was extracted and converted into logOR as the outcome measure. Heterogeneity was assessed using I^2^ statistics, and publication bias using the Fail-Safe N test.

This analysis used the log odds ratio as the outcome measure. A random-effects model was fitted to the data. The amount of heterogeneity (i.e., tau^2^) was estimated using the restricted maximum-likelihood estimator [25]. Along with the tau^2^ estimate, the Q-test for heterogeneity and the I^2^ statistic are also reported [26]. If any heterogeneity is detected (i.e., tau^2^ > 0, regardless of the Q-test results), a prediction interval for the true outcomes is provided. Studentized residuals and Cook’s distances are used to identify studies that may be outliers or influential in the model. Studies with a studentized residual larger than the 100 × (1 − 0.05/(2 × k))th percentile of a standard normal distribution are considered potential outliers (applying a Bonferroni correction with a two-sided alpha of 0.05 for k studies included in the meta-analysis). Studies with a Cook’s distance larger than the median plus six times the interquartile range of Cook’s distances are considered influential. The rank correlation test and the regression test, using the standard error of observed outcomes as a predictor, assess funnel plot asymmetry. For the secondary endpoint, a mixed-effects model was used, and hazard ratios (HR) and 95% confidence intervals (CI) were extracted and converted into logHR and their standard errors to calculate the Summary HR (SHR). Heterogeneity was evaluated with I^2^ statistics, and publication bias was examined using Egger’s regression test. The data analyses were conducted using Jamovi 2.4.11.

## 3. Results

Overall, 30 studies were retrieved, of which 9 were eligible for this meta-analysis after excluding studies involving luminal and HER2+ BC subtypes and those without available data.

**Figure 2:**
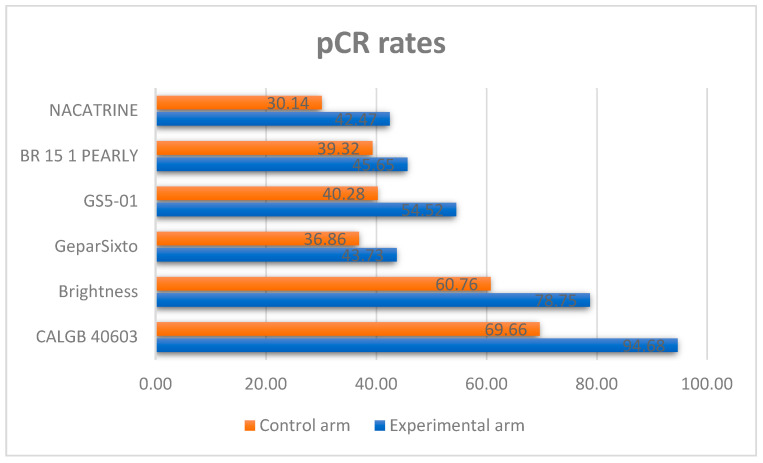
pCR rates in each study included in the meta-analysis.

Of these 9, excluding studies with diverse standard-of-care (SoC) treatment chosen into the control arm and short follow-up (inferior to 4 years), six studies (BrighTNess [27], GeparSixto [28], GS5-01 [29], BR 15 1 PEARLY [30], NACATRINE [31], CALGB 40603 [32]) completely satisfied the inclusion criteria and were included in our investigation with a total of 3402 patients. Table 1 summarizes the main characteristics of each study included in the meta-analysis. Moreover, the pCR rates in the experimental and control arms of each study are shown in Figure 2.

### 3.1. Evaluation of Adding Carboplatin to NACT in Early TNBC on pCR Rate

The addition of CBDCA to NACT was associated with a significant improvement in pCR rate: OR 1.63 [95% CI: 1.38–1.92, *p* < 0.001]; not significant heterogeneity (I^2^ = 0.81%) and without publication bias (Regression Test Z 0.33, *p* = 0.7; Kendal’s Tau 0.067, *p* = 1; Fail-Safe N = 66.000, *p* < 0.001).

A total of six studies were included in the analysis. The observed log odds ratios ranged from 0.2597 to 0.8727, with all estimates being positive (100%). The mean log odds ratio, based on the random-effects model, was 0.4861 (95% CI: 0.3212 to 0.6511). This indicates that the average outcome significantly differs from zero (5.7762, *p* < 0.0001). The Q-test showed no significant heterogeneity among the true outcomes (Q (5) = 4.9046, *p* = 0.4276, tau b2 = 0.0004, I b2 = 0.8087%). The 95% prediction interval for the true outcomes is from 0.3170 to 0.6552. Therefore, although some heterogeneity might exist, the true effects across studies generally point in the same direction as the estimated average. Analyzing the studentized residuals revealed that no study’s value exceeded 2.6383, indicating no outliers in this model. Based on Cook’s distances, no study was overly influential. Additionally, both the rank correlation and regression tests showed no funnel plot asymmetry (*p* = 1.0000 and *p* = 0.7353, respectively).

**Figure 3:**
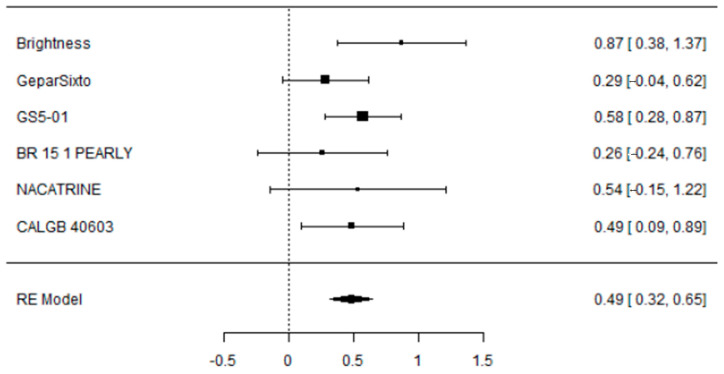
Forest plot of the meta-analysis on percentage of pCR in eTNBC receiving NACT with/without platinum.

**Figure 4:**
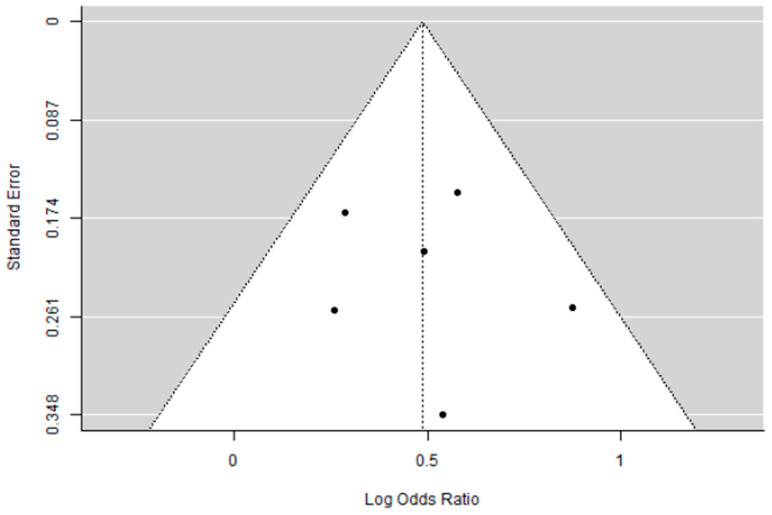
Funnel plot of the meta-analysis on percentage of pCR in eTNBC receiving NACT with/without platinum.

Forest and Funnel plots are reported below as Figure 3 and Figure 4.

### 3.2. Evaluation of Adding Carboplatin to NACT in Early TNBC on DFS Rate

To evaluate the impact of adding carboplatin to NACT in eTNBC patients on disease-free survival (DFS), we performed a primary analysis using a random-effects model with the DerSimonian–Laird estimator. Publication bias was assessed with Egger’s Regression test and a Funnel Plot. An equivalence test was employed to determine whether the results are applicable to the general population.

For this analysis, six studies were considered, each given the same weight in the assessment (no use of moderators).

Adding carboplatin to NACT in eTNBC patients has a positive impact on DFS (SHR = 0.81 [95% CI: 0.63–0.91], *p* = 0.003), with low heterogeneity (I^2^ = 27.95%) and absence of publication bias (Egger’s Regression = 0.006, *p* = 0.995).

To evaluate the actual effect and, consequently, the clinical relevance and applicability of these results to the general population, an equivalence test was performed, yielding a significant result (Z = 2.36, *p* = 0.009), confirming that the patient population included in the meta-analysis is representative of the general population.

**Figure 5:**
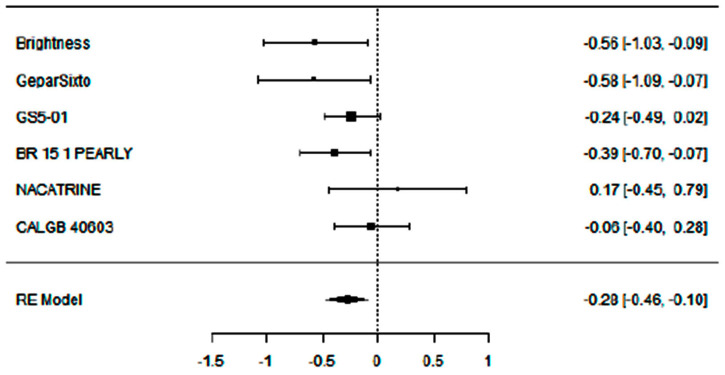
Forest plot of the meta-analysis on DFS rate in eTNBC receiving NACT with/without platinum.

**Figure 6:**
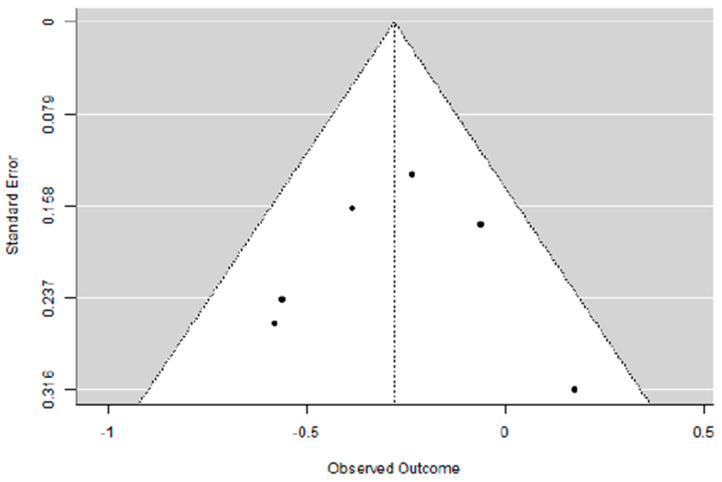
Funnel plot of the meta-analysis on DFS rate in eTNBC receiving NACT with/without platinum.

**Figure 7:**
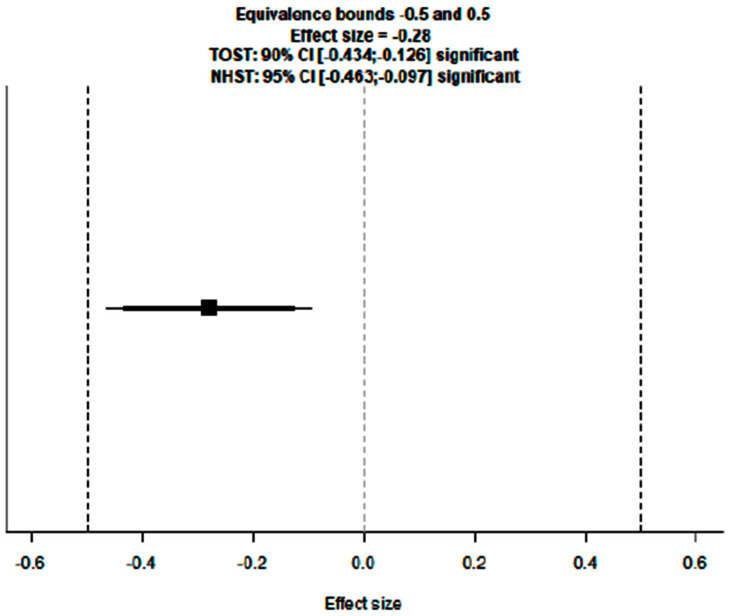
Equivalence test plot of the meta-analysis on DFS rate in eTNBC receiving NACT with/without platinum.

Figure 5, Figure 6 and Figure 7 show, respectively, the Forest Plot, Funnel Plot, and Equivalence Test Plot.

### 3.3. Descriptive Overview of Hematological Toxicity Among Studies

**Figure 8:**
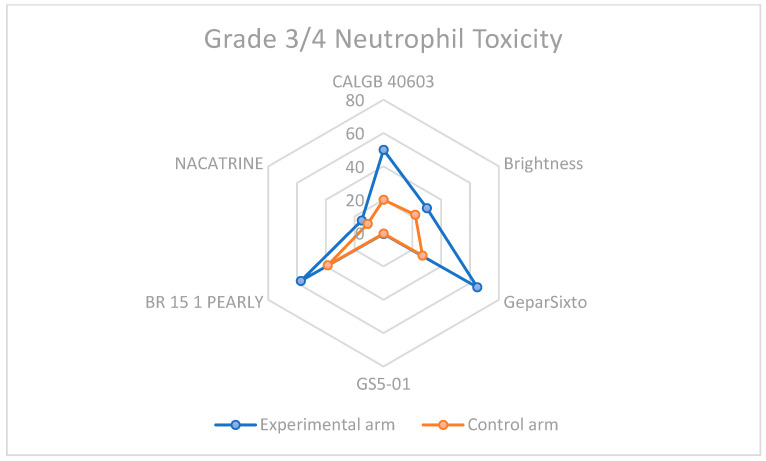
Neutrophil toxicity in grade 3–4 among carboplatin study arms and control groups.

**Figure 9:**
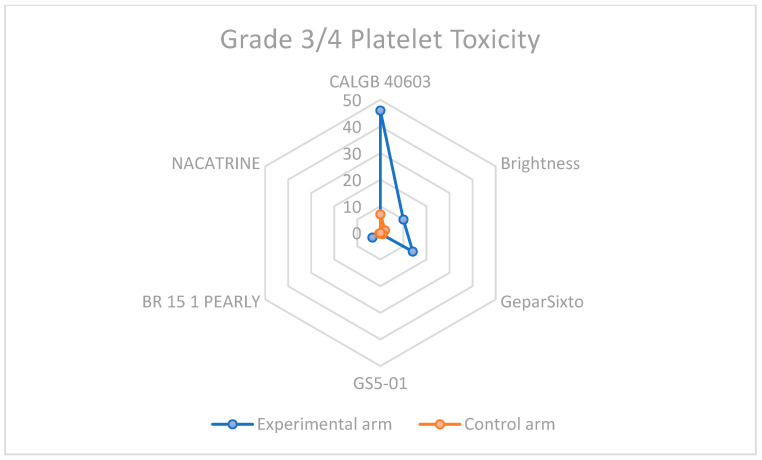
Grade 3–4 platelet toxicity in carboplatin-containing study arms and control arms.

Given that the addition of Carboplatin to neoadjuvant chemotherapy is a winning strategy for eTNBC patients, the increased toxicity, especially hematological, cannot be ignored. In every study considered for the meta-analysis, this type of toxicity was more frequent, as shown in Table 2 and displayed in Figure 8 and Figure 9, highlighting the importance of considering this adverse event when selecting the patient treatment plan.

### 3.4. Supplementary Sensitivity Analysis Including All Eligible Trials

To provide a more comprehensive overview of the available evidence, a supplementary sensitivity analysis including all nine eligible randomized trials was conducted. This analysis confirmed a trend toward better outcomes with the addition of carboplatin, showing a SHR of 0.78 (95% CI 0.60–1.00, *p* = 0.05), although with moderate heterogeneity (I^2^ = 54.4%) and evidence of publication bias.

The increased heterogeneity was mainly driven by the inclusion of three additional phase II studies characterized by methodological limitations. The trial by Zhang et al. [3]. compared carboplatin plus paclitaxel to epirubicin plus paclitaxel in early TNBC and reported higher pCR and EFS rates in the carboplatin arm. However, interpreting these results is limited by the small sample size, overrepresentation of tumors with high Ki-67 (>20%), lower nodal involvement, and the lack of BRCA mutation data, which is relevant for platinum sensitivity. Similarly, the phase II trial by Iwase et al. evaluated the addition of carboplatin to FEC (5-fluorouracil, epirubicin, and cyclophosphamide) followed by a taxane, but was affected by a small sample size, a high proportion of censored patients (~15%) at survival analysis, and the absence of BRCA and post-surgery treatment information. The NEOCART study, comparing docetaxel plus carboplatin to epirubicin/cyclophosphamide followed by a taxane, was not included in the main analysis because of a short follow-up and small sample size, which could affect survival endpoints. Nonetheless, NEOCART provided relevant exploratory insights by reporting HRD-stratified outcomes and supporting the concept of increased platinum sensitivity in germline BRCA pathogenic variant carriers in early-stage disease.

Overall, including these smaller phase II trials slightly decreased the accuracy and reliability of pooled estimates, emphasizing the need for large, well-designed randomized studies with standardized biomarker assessment to clearly determine the long-term effects of carboplatin in early TNBC.

The plots are included in the Supplementary Material [33,34,35].

## 4. Discussion

This meta-analysis shows that adding carboplatin to standard anthracycline–taxane neoadjuvant chemotherapy significantly increases pathologic complete response and disease-free survival in patients with early-stage triple-negative breast cancer, with minimal heterogeneity across randomized clinical trials. These results support continuing to include carboplatin in neoadjuvant treatment plans, especially for patients with high-risk or immunologically “cold” tumors, in whom the absolute benefit may be most significant. Achieving a pathologic complete response consistently links to improved event-free and overall survival at the patient level. However, the CTNeoBC pooled analysis found that pCR cannot universally act as a surrogate endpoint for survival across all breast cancer subtypes, since trial-level correlations are only modest. The U.S. Food and Drug Administration has recognized that improvements in pCR may be “reasonably likely to predict long-term benefit” in high-risk early breast cancer, endorsing its use for accelerated approval in this context. More recent systematic reviews have reinforced the prognostic value of pCR, but not its surrogate value, highlighting the need for confirmatory long-term data.

Although carboplatin increases hematologic toxicity, mainly neutropenia and thrombocytopenia, it may require occasional dose adjustments or delays. As reported in the results section, this type of toxicity becomes more significant in clinical practice because of the risk of delaying doses, reducing dosing schedules, or discontinuation. The data regarding the discontinuation rate suggest an increase in discontinuation NACT around 10% for the GeparSixto trial, while in the other studies, the management of hematological toxicities didn’t always lead to early discontinuation, but sometimes involved changes in the schedules.

Furthermore, anthracycline-sparing combinations such as paclitaxel–carboplatin could provide long-term benefits in cardiac safety and reduce cumulative toxicity, especially in younger or comorbid patients. Ongoing trials are examining alternative dosing schedules to improve tolerability without compromising efficacy. Overall, these findings support the importance of carboplatin intensification in early TNBC, while highlighting the need to balance efficacy with toxicity and to validate surrogate endpoints for long-term outcomes. In the era of chemo-immunotherapy, carboplatin may enhance immunogenic cell death and improve the efficacy of immune checkpoint inhibitors, supporting its continued role in pembrolizumab-based neoadjuvant strategies. Future biomarker-driven, adaptive clinical trials incorporating genomic instability signatures, immune microenvironment profiling, and circulating tumor DNA dynamics are necessary to improve patient selection and determine the best use of carboplatin in early-stage TNBC.

Although carboplatin consistently increases pCR rates in early TNBC, its survival benefit is not uniform across all patients. A significant challenge, therefore, lies in identifying biological or clinical factors that predict which subgroups truly derive benefit from the addition of platinum. In the context of the new standard of neoadjuvant treatment for early TNBC, based on chemo-immunotherapy as per the results of the KEYNOTE-522 study, biomarker and genomic profiling are required to individualize the treatment strategy. Indeed, integrating genomic, immune, and clinicopathologic parameters is essential for tailoring treatment intensity and identifying patients who genuinely benefit from carboplatin intensification.

Among the most extensively studied biomarkers are BRCA1/2 mutations and homologous recombination deficiency (HRD). Carboplatin has been shown to improve pCR rates in early TNBC; however, this benefit appears to be independent of BRCA1/2 or HRD status. Across randomized trials such as GeparSixto, patients carrying germline BRCA1/2 pathogenic variants achieved substantially higher baseline pCR rates, even in the absence of carboplatin, reflecting their intrinsic chemosensitivity. Conversely, the incremental pCR gain from carboplatin was predominantly observed in BRCA wild-type tumors [36]. Similarly, HRD-positive tumors demonstrated higher overall pCR rates and improved survival outcomes, but no significant interaction between HRD status and carboplatin efficacy was detected [37]. Collectively, these findings suggest that BRCA mutations and HRD status define chemosensitive subgroups biologically, but they do not function as predictive biomarkers for the addition of platinum agents.

Further analyses have investigated potential distinctions between BRCA1- and BRCA2-associated breast cancers. Consistently, BRCA1-mutated tumors exhibit higher pCR rates and superior chemotherapy responsiveness compared with BRCA2-mutated or noncarrier tumors [38,39]. This differential sensitivity likely reflects underlying tumor biology: BRCA1-associated cancers are more frequently triple-negative and characterized by a high proliferative index, genomic instability, and basal-like transcriptional profiles, all features linked to heightened chemosensitivity. In contrast, BRCA2-associated tumors often exhibit luminal characteristics, consistent with the typically less pronounced chemotherapy response observed in HR-positive disease. Consequently, the apparent differences in treatment outcomes between BRCA1 and BRCA2 carriers may be driven more by tumor phenotype than by the specific gene mutation itself.

Beyond DNA repair-related biomarkers, the tumor immune microenvironment has emerged as a key determinant of therapeutic response in TNBC. Accumulating evidence underscores the dual prognostic and predictive relevance of stromal tumor-infiltrating lymphocytes (sTILs) in early-stage disease. Higher sTIL levels have been consistently associated with increased pCR rates and improved long-term outcomes, including event-free and overall survival [40].

Recent real-world and prospective studies confirm that elevated sTIL levels identify immunologically “hot” tumors characterized by robust antitumor activity and enhanced sensitivity to immune checkpoint inhibitors, even in the absence of carboplatin. Conversely, patients with low sTIL levels appear to derive limited benefit from pembrolizumab but may benefit from the addition of carboplatin to anthracycline–taxane regimens [41].

Despite their substantial prognostic value, TILs have not yet been validated as predictive biomarkers to guide treatment selection or platinum use. Current evidence suggests that sTILs and PD-L1 expression may jointly refine therapeutic decision-making, particularly in the era of chemoimmunotherapy. Patients with high sTILs and/or PD-L1-positive tumors appear to derive the most significant benefit from pembrolizumab-containing regimens. In contrast, those with low immune infiltration might require optimization of the chemotherapy backbone, including the addition of platinum, to enhance immunogenicity [41].

Nevertheless, the lack of validated predictive thresholds currently limits their clinical applicability, underscoring the need for biomarker-driven studies that link immune profiling to therapeutic adaptation.

Beyond molecular and immune biomarkers, traditional clinical factors continue to play a central role in risk stratification and treatment decisions for early TNBC. Specifically, tumor stage and nodal status remain among the most powerful prognostic determinants, independently predicting both recurrence risk and long-term survival. Patients presenting with node-positive or locally advanced disease experience lower pCR rates and worse outcomes overall [42]. These observations suggest that platinum sensitivity in TNBC is not confined to a specific clinical phenotype but may represent a broader biological feature of chemosensitive tumors.

In this context, integrating clinicopathologic variables with genomic and immune biomarkers may provide a more comprehensive framework for individualizing therapy intensity. While pembrolizumab combined with chemotherapy has become the new neoadjuvant standard for TNBC, the role of carboplatin within this backbone remains uncertain. The findings of this meta-analysis suggest that carboplatin confers a consistent increase in pCR and DFS, supporting its continued inclusion in the neoadjuvant setting, at least until predictive biomarkers allow for de-escalation in selected subgroups. Future research should prioritize biomarker-driven, adaptive neoadjuvant designs that integrate genomic instability signatures, TIL quantification, and circulating tumor DNA dynamics to optimize patient selection for platinum and immunotherapy combinations.

In the era of pembrolizumab, the precise contribution of carboplatin remains under investigation. Current data suggest that carboplatin may enhance immunogenic cell death and increase antigen presentation, potentially synergizing with immune checkpoint blockade. This mechanistic rationale, together with our observed clinical benefit, supports maintaining carboplatin in the backbone of chemoimmunotherapy regimens until validated predictive biomarkers allow safe omission.

Future biomarker-driven, adaptive neoadjuvant trials integrating genomic instability signatures, immune microenvironment characterization, and circulating tumor DNA dynamics are essential to refine patient selection for platinum-based therapy and to define its optimal role within chemoimmunotherapy regimens.

## 5. Conclusions

This meta-analysis demonstrates that the addition of carboplatin to standard anthracycline–taxane neoadjuvant chemotherapy significantly increases pCR rates and improves disease-free survival in patients with early-stage TNBC, with minimal heterogeneity across randomized trials. These findings support the inclusion of carboplatin in the neoadjuvant regimen, particularly in patients with high-risk or immune-cold tumors, where the benefit may be most pronounced. However, the survival advantage appears modest and may not be universal, highlighting the need for biomarker-driven personalization. Future studies should integrate genomic (HRD, BRCA), immunologic (TILs, PD-L1), and clinicopathologic variables to identify patients who derive the most significant benefit from platinum-based intensification and to define its role within chemo-immunotherapy combinations.

## Figures and Tables

**Table 1:** Included studies characteristics. * ddAC: dose-dense anthracycline and cyclophosphamide.

Study	Publishing Year	Phase	Treatment Arms	CBDCA Schedule	eTNBC Patients (n)	Primary Endpoints	Secondary Endpoints	pCR Definition
Brightness	2022	III R	ddAC *+paclitaxel ddAC+paclitaxel+carboplatin ddAC+paclitaxel/carboplatin+veliparib	AUC 6 every 3 weeks ×4 cycles	634	pCR	EFS, OS	ypT0/is ypN0
GeparSixto	2017	II R	ddAC+paclitaxel+bevacizumab+carboplatin ddAC+paclitaxel+bevacizumab	AUC 1.5 weekly × 12 administrations	588	DFS	pCR, OS	ypT0 ypN0
GS5-01	2023	III R	AC+paclitaxel AC+carboplatin+paclitaxel	AUC 2 weekly × 12 administrations	720	DFS	pCR, OS	ypT0 ypN0
BR15-1 PEARLY	2024	III R	AC+paclitaxel AC+carboplatin+paclitaxel	AUC 5 every 3 weeks × 4 cycles	868	EFS	pCR, OS	ypT0/is ypN0
NACATRINE	2023	II R	docetaxel+carboplatin EC+docetaxel	AUC 1.5 Weekly × 12 administrations	146	pCR	DFS, OS	ypT0ypN0
CALGB 40603	2022	II R	ddAC+carboplatin ddAC+carboplatin+bevacizumab ddAC ddAC+bevacizumab	AUC 6 Every three weeks × 4 cycles	446	pCR	EFS, OS	ypT0/is ypN0

**Table 2:** Percentages of severe toxicities among studies selected for the meta-analysis.

Study	CBDCA Arm/s Neutropenia	Non-CBDCA Arm/s Neutropenia	CBDCA Arm/s Thrombocytopenia	Non-CBDCA Arm/s Thrombocytopenia
CALGB 40603	>50	>20	46	7
Brightness	30	22	10	2
GeparSixto	65	27	14	<1
GS5-01	0.55	0.28	0.27	0.2
BR 15 1 PEARLY	57.4	38.7	3.5	0.5
NACATRINE	15.1	11	0	0

## Data Availability

All data used in this meta-analysis are publicly available in the studies included in the review.

## Supplementary Materials

The following supporting information can be downloaded at: https://www.mdpi.com/article/10.3390/cancers17243961/s1.
