# Supplementary material for: Updating the Role of Carboplatin Added to Neoadjuvant Chemotherapy in Early Triple-Negative Breast Cancer: A Meta-Analysis
Source: Cancers (Basel). 2025 Dec 12;17(24):3961. doi: 10.3390/cancers17243961 (PMC12730687; doi:10.3390/cancers17243961)
Supplement: Supplementary file 1 [file cancers-17-03961-s001.zip › cancers-4011036-supplementary.pdf]

Supplementary Material

Effect Sizes and (Sampling Variances or Standard Errors)

Random-Effects Model (k = 9)

	Estimate	se	Z	p	CI Lower Bound	CI Upper Bound
Intercept	-0.245	0.125	-1.96	0.050	-0.490	-0.000
	.	.	.	.	.	.

Nota. Tau^2 Estimator: DerSimonian–Laird

[40]

Heterogeneity Statistics

Tau	Tau^2	I^2	H^2	R^2	df	Q	p
0.254	0.0646 (SE= 0.0657)	54.37%	2.192	.	8.000	17.534	0.025

Forest Plot

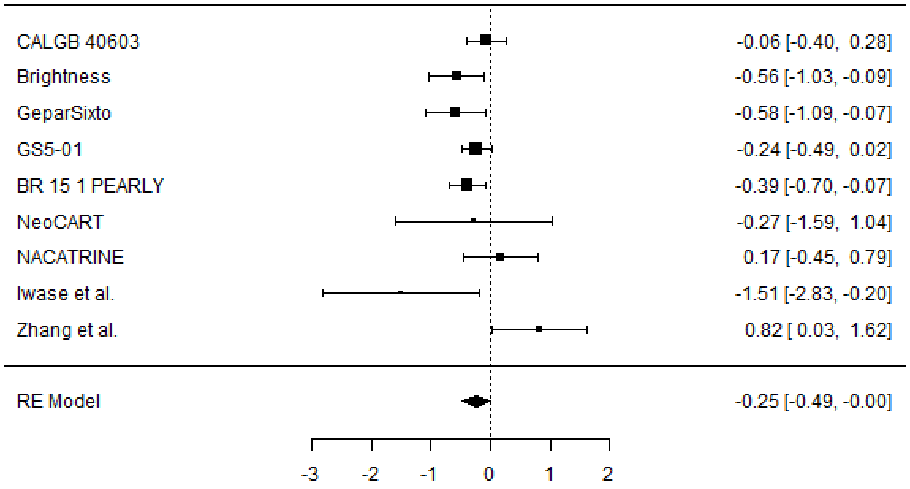

[41]

Publication Bias Assessment

Test Name	value	p
Fail-Safe N	23.000	0.001
Kendalls Tau	0.000	1.000
Egger's Regression	-0.195	0.845

Nota. Fail-safe N Calculation Using the Rosenthal Approach

Funnel Plot

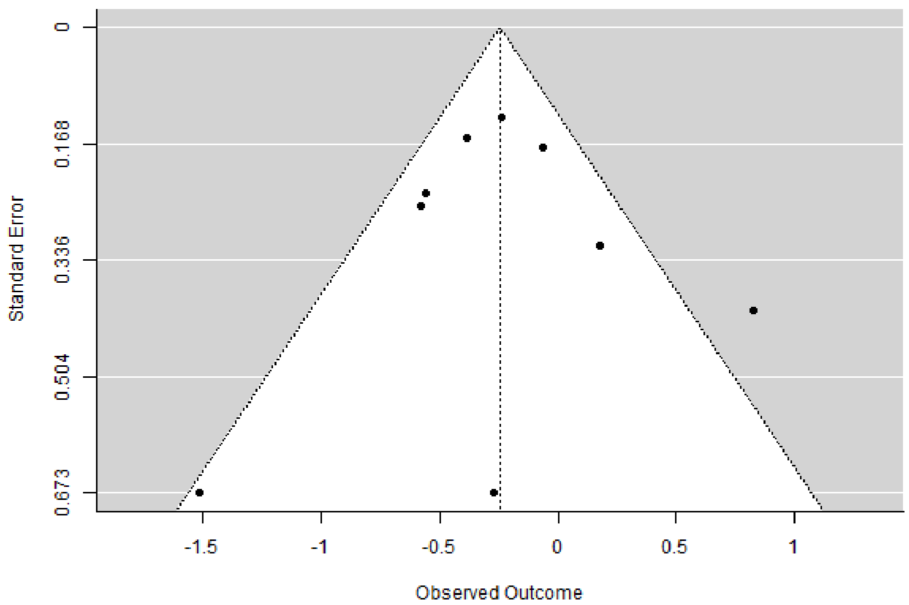

[41]

Two One-Sided Equivalence Tests

Z-Value Lower Bound	P-Value Lower Bound	Z-Value Upper Bound	P-Value Upper Bound	LL_CI_TOST	UL_CI_TOST	LL_CI_ZTEST	UL_CI_ZTEST
2.040	0.021	-5.961	0.000	-0.451	< .001	-0.490	-0.000

[42]

Two One-Sided Equivalence Tests: Text Summary

The equivalence test was significant, Z = 2.040, p = 0.0207, given equivalence bounds of -0.500 and 0.500 and an alpha of 0.05. The null hypothesis test was significant, Z = -1.961, p = 0.0499, given an alpha of 0.05.
NA

Equivalence Test Plot

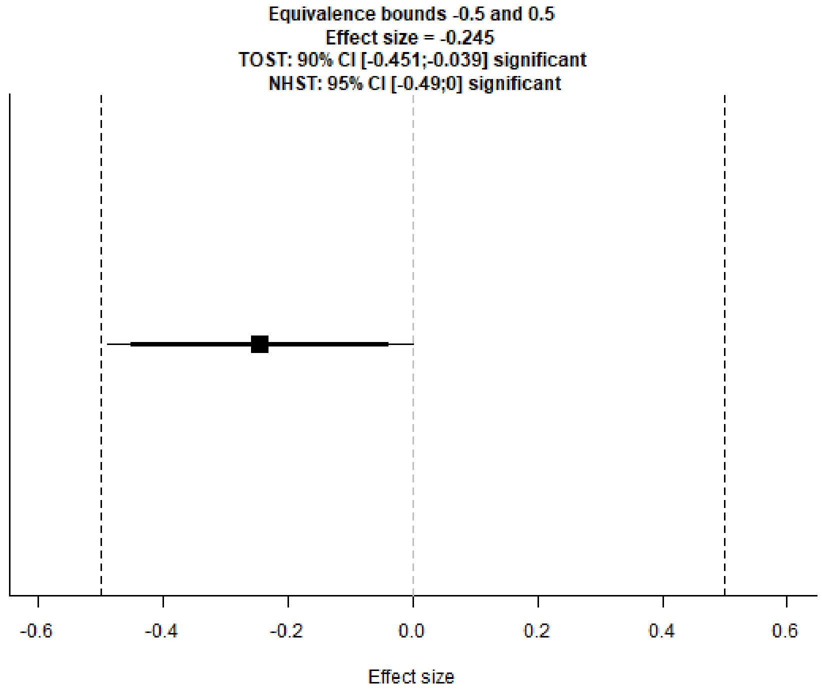

[42]
